# The role of oligodendroglial dysfunction in Huntington's disease

**DOI:** 10.1177/18796397251358017

**Published:** 2025-08-07

**Authors:** Xinhui Li, Shihua Li, Xiao-Jiang Li, Huu Phuc Nguyen, Asa Petersen, Mahmoud A Pouladi

**Affiliations:** 1 Guangdong Provincial Key Laboratory of Non-Human Primate Research, Key Laboratory of CNS Regeneration (Ministry of Education), Guangdong-Hongkong-Macau Institute of CNS Regeneration, Jinan University,Guangzhou, China; 2 Department of Human Genetics, Medical Faculty, 9142 Ruhr University of Bochum, Bochum, Germany; 3 Translational Neuroendocrine Research Unit, Department of Experimental Medical Science, 5193 Lund University, Lund, Sweden; 4 Department of Psychiatry, Skåne University Hospital, Lund, Sweden; 5 Department of Medical Genetics, Centre for Molecular Medicine and Therapeutics, Djavad Mowafaghian Centre for Brain Health, Edwin S. H. Leong Centre for Healthy Aging, 8166 University of British Columbia, British Columbia Children's Hospital Research Institute, Vancouver, BC, Canada

**Keywords:** Glia, myelination, oligodendrocytes, Huntington's disease, white matter

## Abstract

Huntington's disease (HD) is a fatal neurodegenerative disorder characterized by progressive motor, cognitive, and psychiatric symptoms. Research efforts to understand and treat the disease have historically focused on neuronal pathology, but growing evidence underscores the critical role of oligodendrocytes in its pathogenesis. This review synthesizes recent findings on oligodendroglial dysfunction in HD, showing that white matter abnormalities arise early in disease progression, often preceding gray matter changes and clinical symptoms. Neuroimaging and postmortem studies reveal significant white matter atrophy, myelin breakdown, and impaired oligodendrocyte maturation in both patients and animal models. The myelination response to environmental factors is also altered in HD, suggesting impaired white matter plasticity in the disease. At the molecular level, mutant huntingtin disrupts oligodendrocyte function through transcriptional dysregulation of myelin genes, epigenetic modifications involving PRC2 and REST, altered lipid metabolism, thiamine pathway dysfunction, and aberrant BDNF signaling. Key oligodendroglial transcriptional regulators such as MYRF and TCF7L2 are compromised in HD, leading to defective myelination and reduced metabolic support for neurons. Recognizing the role of these mechanisms provides potential biomarkers for early detection and therapeutic targets aimed at preserving both neuronal and glial function in HD.

## Introduction

Huntington's disease (HD) is an autosomal dominant neurodegenerative disorder caused by an expanded CAG repeat within the *HTT* gene, leading to an elongated polyglutamine tract in the huntingtin protein. Clinically, HD is characterized by progressive motor dysfunction, cognitive decline, and psychiatric disturbances. Although traditionally considered neuron-centric, emerging research highlights crucial roles for glial cells, particularly oligodendrocytes, in HD pathology.

Oligodendrocytes play an essential role in the central nervous system (CNS), contributing not only to structural support but also to neuronal health and function. First identified in 1921 by Pío del Río Hortega,^1^ these cells originate from oligodendrocyte progenitor cells (OPCs), which emerge in multiple waves during embryonic development.^2,3^ OPCs migrate extensively, guided by chemotactic cues such as growth factors, extracellular matrix components, and axonal guidance molecules. These progenitors ultimately populate the CNS, where they differentiate into mature oligodendrocytes. This process ensures the proper distribution of oligodendrocytes, maintaining CNS functionality throughout life. In adulthood, OPCs retain their proliferative and regenerative potential, albeit at a reduced capacity, which allows for continued production of oligodendrocytes under normal and pathological conditions.^4,5^

Once differentiated, oligodendrocytes serve a pivotal function in myelinating axons, a process critical for rapid signal conduction.^6^ By wrapping axons in layers of myelin, oligodendrocytes enable saltatory conduction, reducing the capacitance of the axonal membrane and dramatically increasing conduction velocity. The structure of myelin itself reflects its functionality, being composed predominantly of lipids and specialized proteins such as myelin basic protein (MBP) and proteolipid protein (PLP). These components not only stabilize the myelin sheath but also influence its ability to insulate axons effectively. Myelin formation is not confined to development; it continues into adulthood, where adaptive myelination and remodeling occur in response to neural activity and environmental stimuli.^7,8^ This plasticity is essential for cognitive and motor learning, illustrating how dynamic oligodendrocyte function is integral to CNS adaptability.

Oligodendrocytes do more than insulate axons; they provide critical metabolic and trophic support to neurons.^9,10^ Through the transport of energy substrates such as lactate, oligodendrocytes maintain axonal energy metabolism, ensuring neuronal survival. Additionally, they produce neurotrophic factors such as brain-derived neurotrophic factor (BDNF) and glial-derived neurotrophic factor (GDNF), further underscoring their multifaceted role in sustaining neural networks. The importance of these functions becomes evident in neurodegenerative diseases, where oligodendrocyte dysfunction exacerbates neuronal vulnerability.^11,12^ Studies in conditions such as multiple sclerosis and leukodystrophies highlight how demyelination and impaired oligodendroglial support contribute to axonal degeneration and clinical decline.

In HD, the role of oligodendrocytes is gaining attention.^13^ Recent work suggests that mutant huntingtin (mHTT), the protein responsible for HD, disrupts oligodendrocyte function through multiple mechanisms (detailed below) including transcriptional and epigenetic dysregulation. These disruptions impair the expression of myelin genes, leading to early white matter abnormalities observed in both persons with HD (PwHD) and models of the disease. Such findings emphasize that oligodendrocytes are not passive players in HD but actively contribute to the disease's progression in part by failing to support neuronal health and functionality.

The interplay between oligodendrocytes and neurons is particularly critical in the context of myelin plasticity. This adaptive process allows myelin to remodel in response to changes in neural activity and environmental factors. However, in HD, oligodendroglial plasticity appears compromised,^14–17^ limiting the capacity to repair and adapt neural circuits. Moreover, mHTT disrupts the metabolic support that oligodendrocytes provide to neurons, further contributing to neuronal damage. These deficits underline the broader implications of oligodendrocyte dysfunction in HD and suggest that targeting this dysfunction may offer new therapeutic avenues.

Emerging research highlights the dynamic roles of oligodendrocytes in regulating neural networks beyond their classical functions in myelination.^18^ Their ability to respond to neural activity and environmental changes positions them as central players in CNS homeostasis and adaptability. In HD, understanding how oligodendrocytes contribute to disease pathology opens new possibilities for intervention. By focusing on their transcriptional regulation, myelin production, and metabolic functions, novel strategies may be developed to mitigate neuronal damage and improve clinical outcomes.

Oligodendrocytes are thus indispensable to CNS function, not only through their role in myelination but also as active regulators of neuronal health. Their dysfunction in diseases like HD underscores their importance in maintaining neural integrity. As insights into the biology of oligodendrocytes and their involvement in neurodegeneration deepen, the potential for oligodendroglia-focused therapies grows. These advances hold promise for altering the trajectory of neurodegenerative diseases, including HD, by addressing the critical interplay between glial cells and neurons.

## White matter and myelination abnormalities in HD

**Figure 1.**
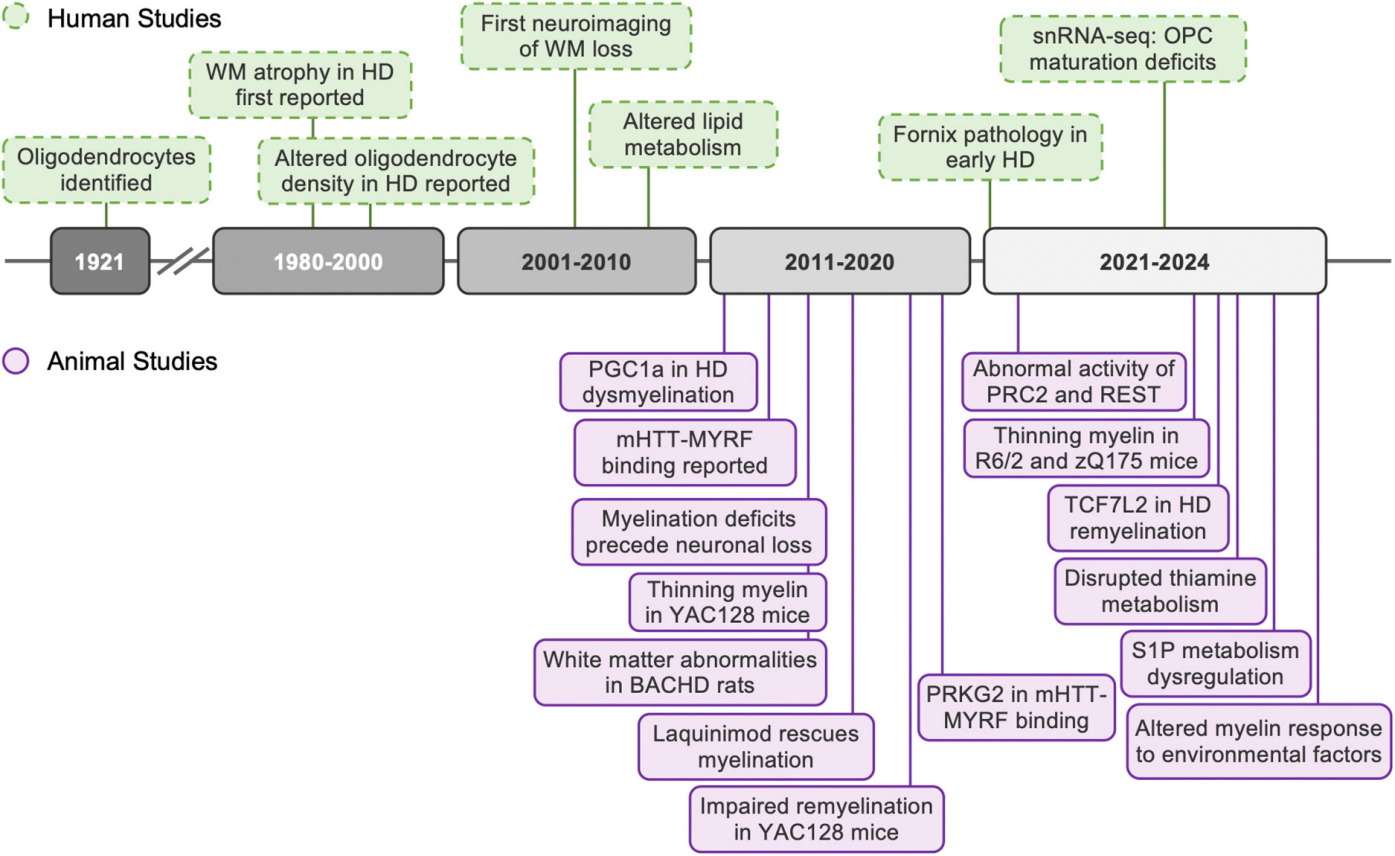
Timeline of notable findings implicating white matter abnormalities and oligodendrocyte pathology in Huntington's disease.

Although mHTT is expressed in all cells in the human body, neuronal cells in the brain are preferentially affected in HD. Emerging studies indicate that glial dysfunction contributes to neuronal damage in HD, with increasing attention focused on the role of oligodendrocytes in disease pathology (Figure 1). Indeed, oligodendrocyte dysregulation is a common and early pathology in the brains of PwHD,^19^ as oligodendrocyte and myelin alterations have been observed in the striatum, corpus callosum, and the fornix of the limbic system of PwHD before onset of motor symptoms.^20–24^

### Evidence from human studies

Through neuroimaging studies, McColgan et al. identified white matter loss in PwHD before motor onset, suggesting that this early impairment may result from metabolic dysregulation and axonal terminal damage caused by toxic mHTT.^22,23^ Changes in white matter including alterations in iron and myelin have been detected as early as 20 years before predicted motor onset in subcortical structures such as the putamen, globus pallidum and the external in the HD-young adult study study.^25^ Furthermore, Gabery et al. reported early pathology in the fornix in the limbic system of patients with HD.^24^ These comprehensive studies employing volumetric analyses of MRI in the IMAGE-HD study demonstrated that the fornix is smaller in PwHD before motor onset compared to controls.^24^ Using postmortem tissue from PwHD, Western blotting analysis confirmed that patients with volume loss of the fornix had lower myelin-regulating factor (MYRF) protein levels compared to non-HD patients.^24^ RNA-seq analysis from HD postmortem tissue further provided a possible mechanistic cause for the white matter damage in the fornix.^24^ The analysis of transcription factor-target gene interactions demonstrated an enrichment of binding sites for the Polycomb Repressive Complex 2 (PRC2) components on their target genes, as well as the level of RE1 Regulation Transcription Factor (REST).^24^ These results suggest that oligodendrocytes in HD suffer from both transcriptional and epigenetic dysregulation at the very early stage induced by mHTT expression directly or indirectly.

Overall, a number of studies of white matter using neuroimaging and analyses of postmortem brain tissue has identified pathology in PwHD. Multiple MRI-based imaging studies in PwHD using both diffusion tensor MRI, neurite orientation dispersion and density imaging (NODDI) and volumetric analyses have demonstrated early and progressive changes in large white matter tracts.^26–29^ Several studies have demonstrated that white matter loss occurs earlier and progresses more rapidly than gray matter loss in PwHD.^30–32^ White matter alterations have been detected in several areas of the brain including in the cortico-basal ganglia white matter tracts, the corpus callosum and the limbic system.^24,25,33–35^

Postmortem analyses of human brain tissue from PwHD have revealed atrophy of white matter^36^ as well as increased density of oligodendrocytes in the striatum.^37^ A recent study of the white matter tract fornix in the limbic system showed atrophy of this region accompanied by ultrastructural and biochemical evidence of damage to myelin.^24^ Bulk and single-cell RNA sequencing of human postmortem tissue from PwHD has shown downregulation of oligodendrocyte identity markers with oligodendrocyte maturation deficits as well as depletion of myelinating oligodendrocytes.^24,38,39^ Taken together, there is now a wealth of studies using both MRI and postmortem analyses of brains from PwHD that show early and progressive alterations in several white matter areas in HD. These changes include atrophy of the tracts as well as myelin breakdown and disruption of the oligodendrocyte maturation transcriptome.

### Evidence from mouse studies

Animal models, particularly rodent models expressing mHTT, have provided crucial insights into oligodendroglial dysfunction in HD (for a detailed review see Ferrari Bardile et al.^13^). Both fragment and full-length models of HD have shown significant white matter abnormalities. Specifically, R6/2 mice (a fragment model) and zQ175 knock-in mice demonstrate reduced myelin thickness, disrupted axonal myelination, and oligodendroglial transcriptional dysregulation.^16,40^ Similarly, electron microscopy analyses in YAC128 mice (expressing full-length mHTT) revealed thinning of myelin sheaths.^14,17,41,42^ These changes parallel the white matter deficits observed in post-mortem HD patient brains.^43^

Furthermore, studies using advanced imaging techniques in rodent models of HD have shown reduced fractional anisotropy (FA), indicative of microstructural changes in white matter tracts; e.g., BACHD rats exhibited significantly lower DT-MRI FA values in the anterior corpus callosum, the cingulum and the external capsule at 12 months of age compared with WT littermates.^41^ These findings suggest myelination deficits in HD and highlight the potential role of oligodendrocytes. mHTT is expressed in these cells, disrupting their function and leading to impaired myelination and altered neuronal metabolic support. Notably, selective expression of mHTT in oligodendrocytes has been shown to result in progressive motor deficits and reduced lifespan in transgenic mice, along with age-dependent demyelination and decreased expression of myelin genes regulated by myelin regulatory factor (MYRF), a transcriptional activator essential for maintaining myelin gene expression in mature oligodendrocytes.^44^ Conversely, inactivating mHTT in oligodendroglia rescues myelin deficits and ameliorates certain behavioral phenotypes in BACHD mice.^45,46^

Oligodendrocyte progenitor cells (OPCs), which are precursors to mature oligodendrocytes, also exhibit dysfunction in HD models. Reduced proliferation and differentiation of OPCs have been reported, potentially contributing to the observed myelination deficits. In the R6/2 model, OPCs display altered morphology and reduced expression of key markers such as Olig2 and Sox10.^38^ Impaired differentiation of OPCs into mature oligodendrocytes results in decreased myelin production and defective repair of demyelinated regions, further exacerbating neuronal vulnerability.

Although this has not been explored in depth to date, the dysfunction of oligodendrocytes in HD is not likely to be solely a cell-autonomous process. Interactions with other glial cells and neurons significantly influence oligodendrocyte health and function.^47^ For example, mHTT expression in neurons can alter axonal signaling, impairing the ability of oligodendrocytes to provide effective myelination. Similarly, astrocytes and microglia are known to play critical roles in oligodendrocyte development and function.^48–50^ Future studies may further clarify these complex astrocyte-microglia-oligodendrocyte interactions and their collective contribution to the myelination abnormalities observed in HD.

## Mechanisms of oligodendrocyte dysfunction

**Figure 2.**
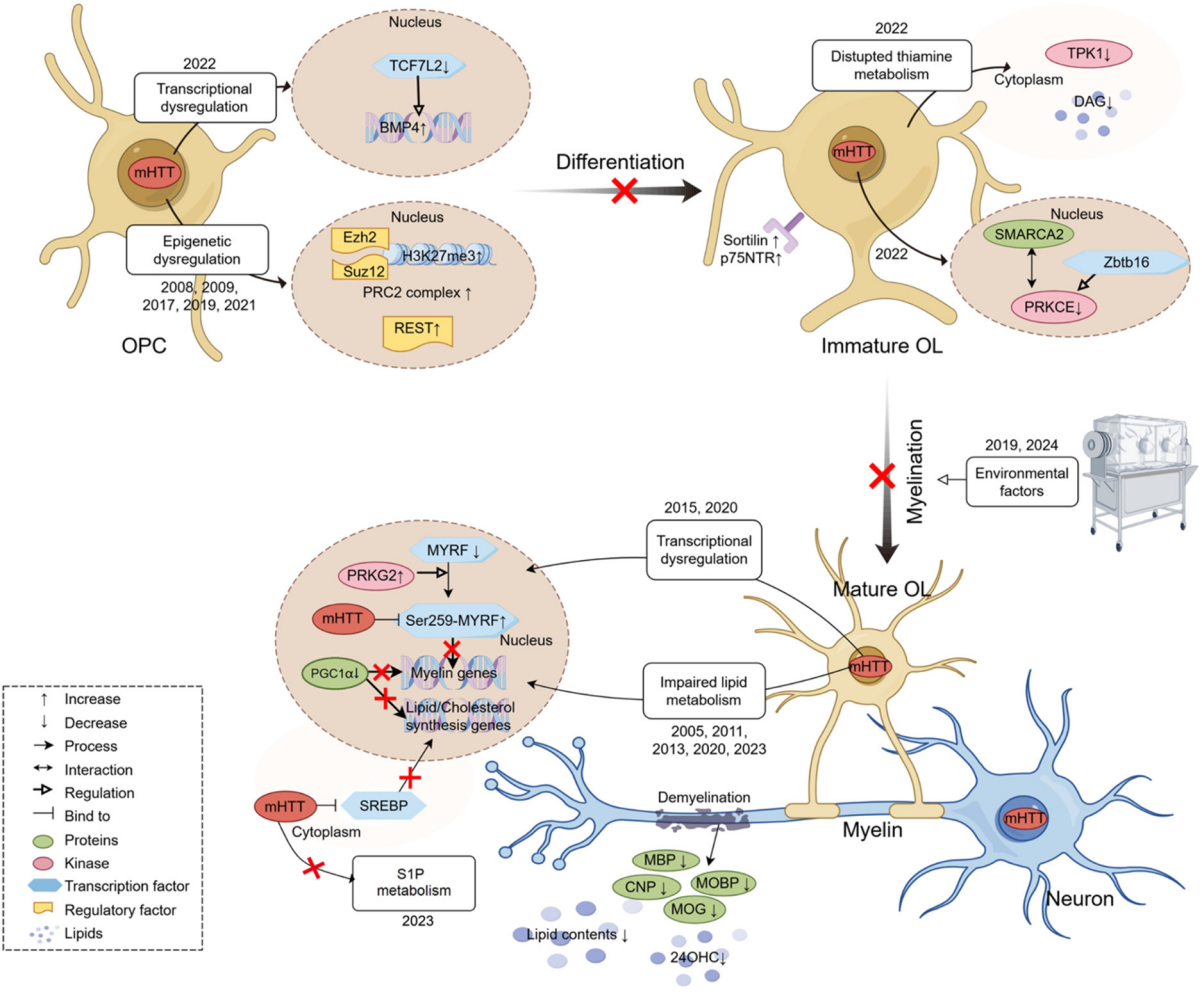
Oligodendrocyte pathogenesis in Huntington's disease. In HD brains, oligodendrocyte pathology arises as early as the stage of oligodendrocyte precursor cells (OPCs) differentiation into immature/mature oligodendrocytes. This process is driven by transcriptional dysregulation, epigenetic remodeling, and disrupted lipid/ sphingosine-1-phosphate (S1P) / thiamine metabolism, and various environmental factors. Ultimately, these alternations lead to myelin-associated protein degradation, lipid loss, and demyelination, impairing neuronal function and integrity. Figure was created using tools on figdraw.com.

Mechanistic studies of white matter pathology in HD have provided insights into how mHTT impacts gene transcription and oligodendrocyte function, implicating several pathways in the pathogenic process (summarized in Figure 2).

*Transcriptional dysregulation.* One key finding is the association of myelination defects with decreased transcription factor 7-like 2 (TCF7L2)-dependent transcriptional pathways.^16^ TCF7L2 plays a crucial role in promoting oligodendrocyte differentiation by tightly regulating bone morphogenetic protein 4 (BMP4) signaling. Reduced TCF7L2 levels lead to increased BMP4 expression in oligodendrocytes, inhibiting the generation and differentiation of oligodendrocytes.^51^

In a mouse model expressing the N-terminal 251 amino acids of mHTT driven by the PLP-promoter (PLP-150Q HD mice), demyelination was observed at 3–5 months.^44^ The demyelination in PLP-150Q mice was attributed to mHTT's abnormal binding to MYRF, affecting its transcriptional activity and reducing the transcription of myelin genes, including MBP, 2′,3′-Cyclic nucleotide 3′-phosphodiesterase (CNP), myelin-associated oligodendrocyte basic protein (MOBP), and myelin oligodendrocyte glycoprotein (MOG).^44^

A subsequent study using laquinimod (LAQ) to treat PLP-150Q HD mice significantly reduced myelin abnormalities by decreasing cGMP-activated protein kinase subunit II (PRKG2). Knocking down PRKG2 using CRISPR/Cas9 further confirmed that abnormal PRKG2 activity enhances mHTT binding to MYRF, affecting its function.^52^ Reduced PRKG2 levels lead to a decrease in phosphorylated Ser259-MYRF, releasing MYRF from the mHTT bond and restoring its normal activity.^52^ The myelin repair effect of LAQ was also demonstrated in YAC128 mice, showing independence from inflammatory regulation, as LAQ acted by regulating inflammation in the treatment of multiple sclerosis.^42^

Furthermore, attenuating mHTT's effects on peroxisome proliferator-activated receptor gamma coactivator 1-alpha (PGC1α), a key regulator of MBP production and cholesterol synthesis in oligodendrocytes, may ameliorate early myelin dysregulation.^53,54^ These studies shed light on the molecular mechanisms underlying white matter damage in HD and suggest potential therapeutic targets for mitigating oligodendrocyte dysfunction and myelin abnormalities in the disease.

*Altered lipid metabolism.* In addition to the mechanisms previously discussed, impaired myelin dysregulation in HD can also be influenced by altered lipid metabolism. Myelin, a lipid-enriched and highly organized multi-layer membrane structure, plays a crucial role in facilitating fast, long-distance saltatory conduction of neuronal impulses.^55^ Animal brain myelination and remyelination rely on fatty acid synthesis in oligodendrocytes.^56^ Several investigators have reported altered lipid metabolism in the brains of individuals with HD.^57,58^

The impaired lipid metabolism in HD may result from reduced biogenesis due to decreased levels of brain-specific catabolite 24S-hydroxycholesterol (24OHC) or due to the binding of mHTT to SREBP, inhibiting its translocation to the nucleus and activating cholesterol and lipid biosynthesis.^59–61^ For example, in 9-month-old LacQ140 HD mice, a decrease in overall striatal lipid content was observed, driven primarily by a reduction in subclasses crucial for white matter integrity, such as ceramide, sphingomyelin, and monogalactosyldiacylglycerol.^62^ Alterations were also noted in phosphatidylinositol, phosphatidylserine, and bismethyl phosphatidic acid.^62^ Transcriptional changes affecting myelin lipids showed partial reversal with reduction of mHTT levels.^62^

Research by Pepe et al. demonstrated that the inhibitor 2-acetyl-5-tetrahydroxybutyl imidazole (THI), an inhibitor of the sphingosine-1-phosphate (S1P) degradative enzyme SGPL1, significantly reduces myelin damage in HD and restores myelin marker proteins in HD models, supporting the dysregulated lipid or fatty acid mechanism in HD.^63,64^ These findings highlight the importance of lipid metabolism in myelin integrity and suggest potential therapeutic targets for addressing myelin impairment in HD through modulation of lipid pathways.

*Epigenetic mechanisms.* Epigenetic dysregulation appears to be another critical mechanism contributing to oligodendrocyte dysfunction in HD. Studies in both human and murine HD models have identified abnormal activity of key epigenetic regulators, including the PRC2 and repressor element-1 silencing transcription factor (REST).^24,45^ Of particular interest is the interaction between mHTT and PRC2, previously shown to enhance PRC2’s histone H3K27 trimethylase activity,^65^ which is reflected in increased H3K27me3 levels in the corpus callosum of BACHD mice.^45^ This heightened PRC2 activity disrupts the transcriptional regulation of myelin-related genes, as evidenced by the increased binding of PRC2 subunits to their regulatory regions.^45^ Key components of the PRC2 complex, such as enhancer of zeste homolog 2 (Ezh2) and suppressor of zeste 12 (Suz12), are essential for OPC differentiation into mature oligodendrocytes by silencing progenitor-specific gene programs.^66,67^ Notably, selective inactivation of mHTT in oligodendroglia of BACHD mice restored normal patterns of Ezh2 and Suz12 binding, further implicating this pathway in oligodendrocyte dysfunction in HD and highlighting its potential as a therapeutic target.^45^

*Role of BDNF and environmental factors.* Although mature BDNF plays a significant neuroprotective role,^68^ its precursors are believed to cause synaptic depression and cell death.^69,70^ A study showed that immature oligodendrocytes in the striatum exhibit markedly elevated levels of the BDNF precursor receptors sortilin and p75NTR, which are believed to be closely associated with HD striatal myelin degradation.^71^

Two recent studies demonstrated that the environment can also influence oligodendrocyte pathology in HD.^15,17^ In a germ-free (GF) environment, mature oligodendrocytes and myelin-associated proteins were reduced in the prefrontal cortex in both wild-type (WT) and BACHD mice. Intriguingly, ultrastructural studies revealed that the GF environment exerted a more pronounced effect on myelin and axons in BACHD mice.^15^ Environmental deprivation experiments demonstrated that myelin sheaths of YAC128 mice were less affected compared to those of wild-type (WT) mice under impoverished housing conditions, suggesting a paradoxical resistance of YAC128 mice to adverse environmental conditions.^17^

*PRKCE and thiamine metabolism.* Recent studies have identified protein kinase C epsilon (PRKCE) and thiamine metabolism as important regulators of oligodendrocyte maturation in HD.^38^ PRKCE, regulated by diacylglycerol (DAG) and the transcriptional factor Zbtb16, interacts with the chromatin remodeler SMARCA2 to influence OPC differentiation. In HD brains and R6/2 mice, reduced PRKCE and DAG levels disrupt oligodendrocyte maturation.^38^ Thiamine metabolism also appears to contribute to oligodendrocyte dysfunction in HD. Thiamine pyrophosphokinase 1 (TPK1) is dysregulated in R6/2 mice, affecting acetyl-CoA and DAG production. Notably, thiamine and biotin supplementation rescued oligodendrocyte maturation deficits in R6/1 HD mice, further supporting a role for this pathway in oligodendrocyte dysfunction in HD.^38^

## Conclusions

Oligodendrocytes contribute significantly to HD pathogenesis through mechanisms extending beyond their classical role in myelination. As evidenced by neuroimaging studies, postmortem analyses, and preclinical models, white matter abnormalities emerge early in disease progression, often preceding gray matter changes. The molecular underpinnings of oligodendrocyte dysfunction in HD involve mHTT-mediated disruption of key transcriptional regulators such as MYRF and TCF7L2, dysregulated lipid and thiamine metabolism, altered BDNF signaling, and epigenetic dysregulation. These mechanisms collectively impair oligodendrocyte differentiation, myelin production, and metabolic support for neurons, exacerbating neuronal vulnerability and disease progression. These molecular disruptions are compounded by the pro-inflammatory environment created by interactions with astrocytes and microglia, which further impair oligodendrocyte health and function.

By recognizing HD as a disease with substantial oligodendroglial involvement, novel therapeutic strategies targeting white matter integrity and oligodendrocyte function can be developed. However, critical knowledge gaps remain. Future research should address the temporal relationship between oligodendrocyte dysfunction and neuronal pathology, regional heterogeneity in oligodendroglial responses, and whether oligodendrocyte-specific interventions can modify disease progression. Furthermore, the interplay between genetic factors and environmental influences on oligodendrocyte health in HD requires further investigation.

Understanding the mechanisms of oligodendrocyte dysfunction in HD may reveal biomarkers for early detection and therapeutic monitoring, potentially leading to combinatorial approaches targeting both neuronal and glial pathologies to effectively alter disease trajectory in PwHD.

## References

[1] Pérez-Cerdá F Sánchez-Gómez MV Matute C . Pío del Río Hortega and the discovery of the oligodendrocytes. Front Neuroanat 2015; 9: 92. 26217196 10.3389/fnana.2015.00092 PMC4493393

[2] van Tilborg E de Theije CGM van Hal M , et al. Origin and dynamics of oligodendrocytes in the developing brain: implications for perinatal white matter injury. Glia 2018; 66: 221–238. 29134703 10.1002/glia.23256 PMC5765410

[3] Kessaris N Fogarty M Iannarelli P , et al. Competing waves of oligodendrocytes in the forebrain and postnatal elimination of an embryonic lineage. Nat Neurosci 2006; 9: 173–179. 16388308 10.1038/nn1620 PMC6328015

[4] Franklin RJM ffrench-Constant C . Regenerating CNS myelin — from mechanisms to experimental medicines. Nat Rev Neurosci 2017; 18: 753–769. 29142295 10.1038/nrn.2017.136

[5] Xin W Chan JR . Myelin plasticity: sculpting circuits in learning and memory. Nat Rev Neurosci 2020; 21: 682–694. 33046886 10.1038/s41583-020-00379-8 PMC8018611

[6] Nave K-A Werner HB . Myelination of the nervous system: mechanisms and functions. Annu Rev Cell Dev Biol 2014; 30: 503–533. 25288117 10.1146/annurev-cellbio-100913-013101

[7] Baraban M Mensch S Lyons DA . Adaptive myelination from fish to man. Brain Res 2016; 1641: 149–161. 26498877 10.1016/j.brainres.2015.10.026 PMC4907128

[8] Nishiyama A Shimizu T Sherafat A , et al. Life-long oligodendrocyte development and plasticity. Semin Cell Dev Biol 2021; 116: 25–37. 33741250 10.1016/j.semcdb.2021.02.004 PMC8292179

[9] Nave K-A . Myelination and the trophic support of long axons. Nat Rev Neurosci 2010; 11: 275–283. 20216548 10.1038/nrn2797

[10] Philips T Rothstein JD . Oligodendroglia: metabolic supporters of neurons. J Clin Investig 2017; 127: 3271–3280. 28862639 10.1172/JCI90610 PMC5669561

[11] Lee Y Morrison BM Li Y , et al. Oligodendroglia metabolically support axons and contribute to neurodegeneration. Nature 2012; 487: 443–448. 22801498 10.1038/nature11314 PMC3408792

[12] Kang SH Li Y Fukaya M , et al. Degeneration and impaired regeneration of gray matter oligodendrocytes in amyotrophic lateral sclerosis. Nat Neurosci 2013; 16: 571–579. 23542689 10.1038/nn.3357 PMC3637847

[13] Bardile CF Radulescu CI Pouladi MA . Oligodendrocyte pathology in Huntington’s disease: from mechanisms to therapeutics. Trends Mol Med 2023; 29: 802–816. 37591764 10.1016/j.molmed.2023.07.010

[14] Teo RTY Bardile CF Tay YL , et al. Impaired remyelination in a mouse model of huntington disease. Mol Neurobiol 2019; 56: 6873–6882. 30937636 10.1007/s12035-019-1579-1

[15] Radulescu CI Garcia-Miralles M Sidik H , et al. Manipulation of microbiota reveals altered callosal myelination and white matter plasticity in a model of Huntington disease. Neurobiol Dis 2019; 127: 65–75. 30802499 10.1016/j.nbd.2019.02.011

[16] Benraiss A Mariani JN Tate A , et al. A TCF7L2-responsive suppression of both homeostatic and compensatory remyelination in Huntington disease mice. Cell Rep 2022; 40: 111291. 36044851 10.1016/j.celrep.2022.111291

[17] Radulescu CI Bardile CF Garcia-Miralles M , et al. Environmental deprivation effects on myelin ultrastructure in huntington disease and wildtype mice. Mol Neurobiol 2024; 61: 4278–4288. 38079108 10.1007/s12035-023-03799-6

[18] Xiao Y Czopka T . Myelination-independent functions of oligodendrocyte precursor cells in health and disease. Nat Neurosci 2023; 26: 1663–1669. 37653126 10.1038/s41593-023-01423-3

[19] Sun Y Tong H Yang T , et al. Insights into white matter defect in huntington’s disease. Cells 2022; 11: 3381. 36359783 10.3390/cells11213381 PMC9656068

[20] Paola MD Phillips OR Sanchez-Castaneda C , et al. MRI Measures of corpus callosum iron and myelin in early huntington’s disease. Hum Brain Mapp 2014; 35: 3143–3151. 24895252 10.1002/hbm.22391 PMC6869772

[21] Fennema-Notestine C Archibald S Jacobson M , et al. In vivo evidence of cerebellar atrophy and cerebral white matter loss in Huntington disease. Neurology 2004; 63(6): 898–995. 10.1212/01.wnl.0000138434.68093.67 15452288

[22] McColgan P Seunarine KK Gregory S , et al. Topological length of white matter connections predicts their rate of atrophy in premanifest Huntington’s disease. JCI Insight 2017; 2: e92641. 10.1172/jci.insight.92641 PMC5396531 28422761

[23] McColgan P Gregory S Seunarine KK , et al. Brain regions showing white matter loss in Huntington’s disease are enriched for synaptic and metabolic genes. Biol Psychiatry 2018; 83: 456–465. 29174593 10.1016/j.biopsych.2017.10.019 PMC5803509

[24] Gabery S Kwa JE Cheong RY , et al. Early white matter pathology in the fornix of the limbic system in Huntington disease. Acta Neuropathol 2021; 142: 791–806. 34448021 10.1007/s00401-021-02362-8 PMC8500909

[25] Johnson EB Parker CS Scahill RI , et al. Altered iron and myelin in premanifest Huntington’s disease more than 20 years before clinical onset: evidence from the cross-sectional HD young adult study. EBioMedicine 2021; 65: 103266. 33706250 10.1016/j.ebiom.2021.103266 PMC7960938

[26] Rosas HD Tuch DS Hevelone ND , et al. Diffusion tensor imaging in presymptomatic and early Huntington’s disease: selective white matter pathology and its relationship to clinical measures. Movement Disord 2006; 21: 1317–1325. 16755582 10.1002/mds.20979

[27] Bohanna I Georgiou-Karistianis N Sritharan A , et al. Diffusion tensor imaging in Huntington’s disease reveals distinct patterns of white matter degeneration associated with motor and cognitive deficits. Brain Imaging Behav 2011; 5: 171–180. 21437574 10.1007/s11682-011-9121-8

[28] Rosas HD Wilkens P Salat DH , et al. Complex spatial and temporally defined myelin and axonal degeneration in Huntington disease. NeuroImage: Clin 2018; 20: 236–242. 30090698 10.1016/j.nicl.2018.01.029 PMC6078048

[29] Zhang J Gregory S Scahill RI , et al. In vivo characterization of white matter pathology in pre-manifest Huntington’s disease. Ann Neurol. 2018; 84(4): 497–504. 30063250 10.1002/ana.25309 PMC6221120

[30] Ciarmiello A Cannella M Lastoria S , et al. Brain white-matter volume loss and glucose hypometabolism precede the clinical symptoms of Huntington’s disease. J Nucl Med: Off Publ, Soc Nucl Med 2006; 47: 215–222. 16455626

[31] Aylward EH Nopoulos PC Ross CA , et al. Longitudinal change in regional brain volumes in prodromal Huntington disease. J Neurol, Neurosurg Psychiatry 2011; 82: 405. 20884680 10.1136/jnnp.2010.208264 PMC3105627

[32] Tabrizi SJ Reilmann R Roos RAC , et al. Potential endpoints for clinical trials in premanifest and early Huntington’s disease in the TRACK-HD study: analysis of 24 month observational data. Lancet Neurol 2012; 11: 42–53. 22137354 10.1016/S1474-4422(11)70263-0

[33] Rosas HD Lee SY Bender AC , et al. Altered white matter microstructure in the corpus callosum in Huntington’s disease: implications for cortical “disconnection.”. Neuroimage 2010; 49: 2995–3004. 19850138 10.1016/j.neuroimage.2009.10.015 PMC3725957

[34] Zeun P McColgan P Dhollander T , et al. Timing of selective basal ganglia white matter loss in premanifest Huntington’s disease. Neuroimage Clin 2022; 33: 102927. 34999565 10.1016/j.nicl.2021.102927 PMC8757039

[35] Dumas EM van den Bogaard SJA Ruber ME , et al. Early changes in white matter pathways of the sensorimotor cortex in premanifest Huntington’s disease. Hum Brain Mapp 2012; 33: 203–212. 21264990 10.1002/hbm.21205 PMC6869939

[36] de la Monte SM Vonsattel J-P Richardson EP . Morphometric demonstration of atrophic changes in the cerebral cortex, white matter, and neostriatum in Huntington’s disease. J Neuropathol Exp Neurol 1988; 47: 516–525. 2971785 10.1097/00005072-198809000-00003

[37] Myers RH Vonsattel JP Paskevich PA , et al. Decreased neuronal and increased oligodendroglial densities in Huntington’s disease caudate nucleus. J Neuropathol Exp Neurol 1991; 50: 729–742. 1836225 10.1097/00005072-199111000-00005

[38] Lim RG Al-Dalahmah O Wu J , et al. Huntington disease oligodendrocyte maturation deficits revealed by single-nucleus RNAseq are rescued by thiamine-biotin supplementation. Nat Commun 2022; 13: 7791. 36543778 10.1038/s41467-022-35388-x PMC9772349

[39] Bøstrand SMK Seeker LA Bestard-Cuche N , et al. Mapping the glial transcriptome in Huntington’s disease using snRNAseq: selective disruption of glial signatures across brain regions. Acta Neuropathol Commun 2024; 12: 165. 39428482 10.1186/s40478-024-01871-3 PMC11492505

[40] Malaiya S Cortes-Gutierrez M Herb BR , et al. Single-Nucleus RNA-Seq reveals dysregulation of striatal cell identity due to Huntington’s disease mutations. J Neurosci 2021; 41: 5534–5552. 34011527 10.1523/JNEUROSCI.2074-20.2021 PMC8221598

[41] Teo RTY Hong X Yu-Taeger L , et al. Structural and molecular myelination deficits occur prior to neuronal loss in the YAC128 and BACHD models of Huntington disease. Hum Mol Genet 2016; 25: 2621–2632. 27126634 10.1093/hmg/ddw122 PMC5181633

[42] Garcia-Miralles M Yusof NABM Tan JY , et al. Laquinimod treatment improves myelination deficits at the transcriptional and ultrastructural levels in the YAC128 mouse model of Huntington disease. Mol Neurobiol 2019; 56: 4464–4478. 30334188 10.1007/s12035-018-1393-1

[43] Halliday GM McRitchie DA Macdonald V , et al. Regional specificity of brain atrophy in Huntington’s disease. Exp Neurol 1998; 154: 663–672. 9878201 10.1006/exnr.1998.6919

[44] Huang B Wei W Wang G , et al. Mutant huntingtin downregulates myelin regulatory factor-mediated myelin gene expression and affects mature oligodendrocytes. Neuron 2015; 85: 1212–1226. 25789755 10.1016/j.neuron.2015.02.026 PMC4366619

[45] Bardile CF Garcia-Miralles M Caron NS , et al. Intrinsic mutant HTT-mediated defects in oligodendroglia cause myelination deficits and behavioral abnormalities in Huntington disease. Proc Natl Acad Sci 2019; 116: 9622–9627. 31015293 10.1073/pnas.1818042116 PMC6511031

[46] Bardile CF Sidik H Quek R , et al. Abnormal spinal cord myelination due to oligodendrocyte dysfunction in a model of Huntington’s disease. J Huntington’s Dis 2021; 10: 377–384. 34366364 10.3233/JHD-210495

[47] Santos EN Fields RD . Regulation of myelination by microglia. Sci Adv 2021; 7: eabk1131. 10.1126/sciadv.abk1131 PMC8664250 34890221

[48] McNamara NB Munro DAD Bestard-Cuche N , et al. Microglia regulate central nervous system myelin growth and integrity. Nature 2023; 613(7942): 120–129. 36517604 10.1038/s41586-022-05534-y PMC9812791

[49] Camargo N Goudriaan A van Deijk A-LF , et al. Oligodendroglial myelination requires astrocyte-derived lipids. PLoS Biol 2017; 15: e1002605. 10.1371/journal.pbio.1002605 PMC5446120 28549068

[50] Dutta DJ Woo DH Lee PR , et al. Regulation of myelin structure and conduction velocity by perinodal astrocytes. Proc Natl Acad Sci 2018; 115: 11832–11837. 30373833 10.1073/pnas.1811013115 PMC6243273

[51] Zhang S Wang Y Zhu X , et al. The wnt effector TCF7l2 promotes oligodendroglial differentiation by repressing autocrine BMP4-mediated signaling. J Neurosci 2021; 41: 1650–1664. 33452226 10.1523/JNEUROSCI.2386-20.2021 PMC8115879

[52] Yin P Liu Q Pan Y , et al. Phosphorylation of myelin regulatory factor by PRKG2 mediates demyelination in Huntington’s disease. EMBO Rep 2020; 21: EMBR201949783. 10.15252/embr.201949783 PMC9336218 32270922

[53] Xiang Z Valenza M Cui L , et al. Peroxisome-proliferator-activated receptor gamma coactivator 1 α contributes to dysmyelination in experimental models of Huntington’s disease. J Neurosci 2011; 31: 9544–9553. 21715619 10.1523/JNEUROSCI.1291-11.2011 PMC3132810

[54] Xiang Z Krainc D . Pharmacological upregulation of PGC1α in oligodendrocytes: implications for Huntington’s disease. J Huntingt’s Dis 2013; 2: 101–105. 10.3233/JHD-130046 25063433

[55] Montani L . Lipids in regulating oligodendrocyte structure and function. Semin Cell Dev Biol 2021; 112: 114–122. 32912639 10.1016/j.semcdb.2020.07.016

[56] Dimas P Montani L Pereira JA , et al. CNS Myelination and remyelination depend on fatty acid synthesis by oligodendrocytes. eLife 2019; 8: e44702. 10.7554/eLife.44702 PMC6504237 31063129

[57] Block RC Dorsey ER Beck CA , et al. Altered cholesterol and fatty acid metabolism in Huntington disease. J Clin Lipidol 2010; 4: 17–23. 20802793 10.1016/j.jacl.2009.11.003 PMC2926984

[58] Phillips GR Hancock SE Jenner AM , et al. Phospholipid profiles are selectively altered in the Putamen and white frontal cortex of Huntington’s disease. Nutrients 2022; 14: 2086. 35631226 10.3390/nu14102086 PMC9143248

[59] Valenza M Cattaneo E . Emerging roles for cholesterol in Huntington’s disease. Trends Neurosci 2011; 34: 474–486. 21774998 10.1016/j.tins.2011.06.005

[60] Valenza M Rigamonti D Goffredo D , et al. Dysfunction of the cholesterol biosynthetic pathway in Huntington’s disease. J Neurosci 2005; 25: 9932–9939. 16251441 10.1523/JNEUROSCI.3355-05.2005 PMC6725556

[61] Pardo AD Monyror J Morales LC , et al. Mutant huntingtin interacts with the sterol regulatory element-binding proteins and impairs their nuclear import. Hum Mol Genet 2020; 29: 418–431. 31875875 10.1093/hmg/ddz298

[62] Shing K Sapp E Boudi A , et al. Early whole-body mutant huntingtin lowering averts changes in proteins and lipids important for synapse function and white matter maintenance in the LacQ140 mouse model. Neurobiol Dis 2023; 187: 106313. 37777020 10.1016/j.nbd.2023.106313 PMC10731584

[63] Pepe G Capocci L Marracino F , et al. Treatment with THI, an inhibitor of sphingosine-1-phosphate lyase, modulates glycosphingolipid metabolism and results therapeutically effective in experimental models of Huntington’s disease. Mol Ther 2023; 31: 282–299. 36116006 10.1016/j.ymthe.2022.09.004 PMC9840122

[64] Pepe G Lenzi P Capocci L , et al. Treatment with the glycosphingolipid modulator THI rescues myelin integrity in the Striatum of R6/2 HD mice. Int J Mol Sci 2023; 24: 5956. 36983032 10.3390/ijms24065956 PMC10053002

[65] Seong I Woda J Song J , et al. Huntingtin facilitates polycomb repressive complex 2. Epub ahead of print December 3, 2009. DOI: 10.1093/hmg/ddp524. PMC2807366 19933700

[66] Sher F Rössler R Brouwer N , et al. Differentiation of neural stem cells into oligodendrocytes: involvement of the polycomb group protein Ezh2. Stem Cells (Dayton, Ohio) 2008; 26: 2875–2883. 18687996 10.1634/stemcells.2008-0121

[67] He D Wang J Lu Y , et al. lncRNA functional networks in oligodendrocytes reveal stage-specific myelination control by an lncOL1/Suz12 Complex in the CNS. Neuron 2017; 93: 362–378. 28041882 10.1016/j.neuron.2016.11.044 PMC5600615

[68] Miranda-Lourenço C Ribeiro-Rodrigues L Fonseca-Gomes J , et al. Challenges of BDNF-based therapies: from common to rare diseases. Pharmacol Res 2020; 162: 105281. 33161136 10.1016/j.phrs.2020.105281

[69] Vincenti APD Ríos AS Paratcha G , et al. Mechanisms that modulate and diversify BDNF functions: implications for hippocampal synaptic plasticity. Front Cell Neurosci 2019; 13: 135. 31024262 10.3389/fncel.2019.00135 PMC6465932

[70] Chen Z Tang H-B Kang J-J , et al. Necroptotic astrocytes induced neuronal apoptosis partially through EVs-derived pro-BDNF. Brain Res Bull 2021; 177: 73–80. 34555432 10.1016/j.brainresbull.2021.09.014

[71] Ma Q Yang J Li T , et al. Selective reduction of striatal mature BDNF without induction of proBDNF in the zQ175 mouse model of Huntington’s disease. Neurobiol Dis 2015; 82: 466–477. 26282324 10.1016/j.nbd.2015.08.008 PMC4819334

